# Evaluation of retinal and choroidal variations in thyroid-associated ophthalmopathy using optical coherence tomography angiography

**DOI:** 10.1186/s12886-020-01692-7

**Published:** 2020-10-20

**Authors:** Lanchu Yu, Qin Jiao, Yu Cheng, Yanji Zhu, Zhongjing Lin, Xi Shen

**Affiliations:** 1 Department of Ophthalmology, Rui Jin Hospital, School of Medicine, Shanghai Jiao Tong University, 197 Rui Jin Er Road, Shanghai, 200025 China; 2 Department of Ophthalmology, Ren Ji Hospital, School of Medicine, Renji Hospital Affiliated Medical School, Shanghai Jiao Tong University, 160 Pu Jian Road, Shanghai, 200127 China

**Keywords:** Thyroid-associated ophthalmopathy, Optical coherence tomography angiography, Choroidal thickness, Retinal nerve fiber layer

## Abstract

**Background:**

To investigate the difference in retinal nerve fiber layer (RNFL) thickness, choroidal thickness (CT) and superficial retinal vessels between thyroid-associated ophthalmopathy (TAO) patients and healthy controls. To identify the potential influencing factors for these parameters and evaluate their diagnostic abilities in TAO.

**Methods:**

Twenty active TAO patients, 33 inactive TAO patients and 29 healthy participants were enrolled. TAO patients were divided according to the clinical activity score (CAS). RNFL thickness and CT were measured by HD-OCT, while foveal avascular zone (FAZ), vascular density and perfusion density were measured by optical coherence tomography angiography (OCTA). SPSS software was used for statistical analysis.

**Results:**

Active TAO patients had thinner RNFL thickness than the other two groups (*P* < 0.001, *P* < 0.001). Both active and inactive TAO patients had significantly higher CT in the macular region (all *P* < 0.05). The FAZ area in the active TAO group was significantly larger than the other two groups (*P* = 0.045, *P* = 0.001). The inactive TAO group had significantly higher vascular density than the other two groups (all *P* < 0.05). With regard to the perfusion density, significant differences were observed in the temporal and inferior areas (*P* = 0.045, *P* = 0.001), as well as the average values (*P* = 0.032). The FAZ area was positively correlated with intraocular pressure (*r* = 0.274, *P* = 0.013), while it was negatively correlated with axial length (*r* = − 0.344, *P* = 0.002). The vascular density and perfusion density were not significantly correlated with different clinical variables (all *P* > 0.05). The AUC analysis indicated these parameters also exhibited a significant discriminatory power in TAO diagnosis.

**Conclusions:**

TAO patients had significant variations in RNFL thickness, choroidal thickness, FAZ area and superficial retinal vessels. These parameters appeared to be potential adjuncts in the evaluation of TAO patients.

## Background

Thyroid-associated ophthalmopathy (TAO) is a systemic autoimmune disorder or an organ-specific autoimmune inflammatory disease of orbital tissues. It is characterized by inflammatory cellular infiltration with lymphocytes, plasma cells, macrophages and mast cells. The most obvious pathological changes in orbital tissues include interstitial tissue edema, orbital fat hyperplasia and massively swollen extraocular muscles, which may cause orbital compression symptoms [1–4]. Approximately 20% of patients with immune thyroid diseases will develop TAO, and 25–50% of TAO cases are closely related to hyperthyroidism, commonly termed as Graves’ ophthalmopathy, with higher morbidity in females [3, 4]. The exact pathogenesis of TAO remains unknown, but the clinical manifestations can be explained by the expansion of orbital volume due to autoimmune inflammatory infiltration. TAO can be classified into the following phases: an active phase with rapid progression and an inactive phase with symptom stabilization [5, 6]. However, approximately 3–5% of TAO patients in an inactive state will transit to an active state, which may result in aggravation of proptosis, lid retraction, dysfunctional eye motility, or even vision loss due to optic nerve compression [7, 8].

Different techniques have been employed to analyze the retinal and choroidal changes in TAO patients. Walasik-Szemplińska et al. [9] observed the alterations of ocular blood supply in TAO patients by color Doppler imaging. However, there are several influencing factors in the measuring process of ocular blood flow, such as eyeball movement and Doppler angles. The diameters of the examined vessels also limit its clinical applications. Optical coherence tomography (OCT) is a non-invasive imaging technique that used to visualize the detailed structures of the retina and choroid. Sayin et al. [10] found TAO patients had thinner inferior retinal nerve fiber layer (RNFL) thickness and macular thickness. Furthermore, choroidal thickness (CT) was found to be increased in TAO patients [11, 12]. With the advancement in technology, OCT angiography (OCTA) is a new promising imaging technique that is capable of imaging the retinal and choroidal vasculature noninvasively [13–15]. Recently, OCTA has been widely used in ophthalmic diseases such as glaucoma and diabetic eye disease. However, few studies have investigated the retinal vessels in the macular region in TAO patients using OCTA.

Currently, the evaluation of TAO has to be done qualitatively according to the clinical activity score (CAS) system*.* If the evaluation can be carried out quantitatively, the disease severity will be determined with high accuracy. Therefore, we aimed to examine the detailed retinal and choroidal variations in patients with active and inactive TAO using OCTA. We also aimed to explore their correlations with different ocular parameters and investigate their clinical diagnostic capability when comparing with healthy controls.

## Methods

### Study population

In our study, all subjects were recruited from the Department of Ophthalmology at Ruijin Hospital, Shanghai Jiao Tong University School of Medicine between December 2015 and December 2017. All participants signed informed consent forms to participate in the study and received immediate medical attention when needed.

TAO patients were divided into two groups according to the CAS [16]: (1) spontaneous orbital pain, (2) gaze-evoked orbital pain, (3) eyelid swelling, (4) eyelid erythema, (5) conjunctival redness, (6) chemosis, and (7) caruncle inflammation or plica. Patients with CAS ≥ 3/7 were classified as active TAO, and those with CAS ≤ 2/7 were classified as inactive TAO. All TAO patients were newly diagnosed. Age-matched healthy volunteers seeking physical examinations were enrolled as the control group in the same time period. The exclusion criteria for both patients and healthy participants were as follows: (1) special treatment for thyroid diseases within 3 months, such as radioactive iodine therapy, immunosuppressor agents and thyroid surgery; (2) hormonotherapy within 6 months; (3) history of ocular surgical procedures; (4) concomitant ocular diseases, such as glaucoma, retinal vein occlusion, and maculopathy; (5) concurrent infection or severe systemic diseases.

All participants underwent a complete ophthalmic examination including best-corrected visual acuity, intraocular pressure (IOP) measurement with Goldmann applanation tonometry, slit lamp examination and fundus examination. B-scan ultrasonography was performed to assess the ocular and orbital structure. Central corneal thickness (CCT) and axial length (AL) were recorded using Lenstar LS900 (Haag-Streit AG, Switzerland). The proptosis was measured by the same examiner who was experienced at Hertel exophthalmometry.

### Image acquisition and processing

All participants were examined with a traditional high-definition OCT system (Carl Zeiss Meditec, Dublin, CA, USA). The optic disc cube 200 × 200 mode was conducted to obtain RNFL results. CT measurements were performed in the superior, inferior, nasal and temporal regions with selected locations, including 500 μm, 1000 μm, 1500 μm, and 2000 μm from the fovea. CT was defined as the distance between the hyper-reflective line of Bruch’s membrane and the innermost hyper-reflective line of the choroidoscleral interface [17].

**Fig. 1:**
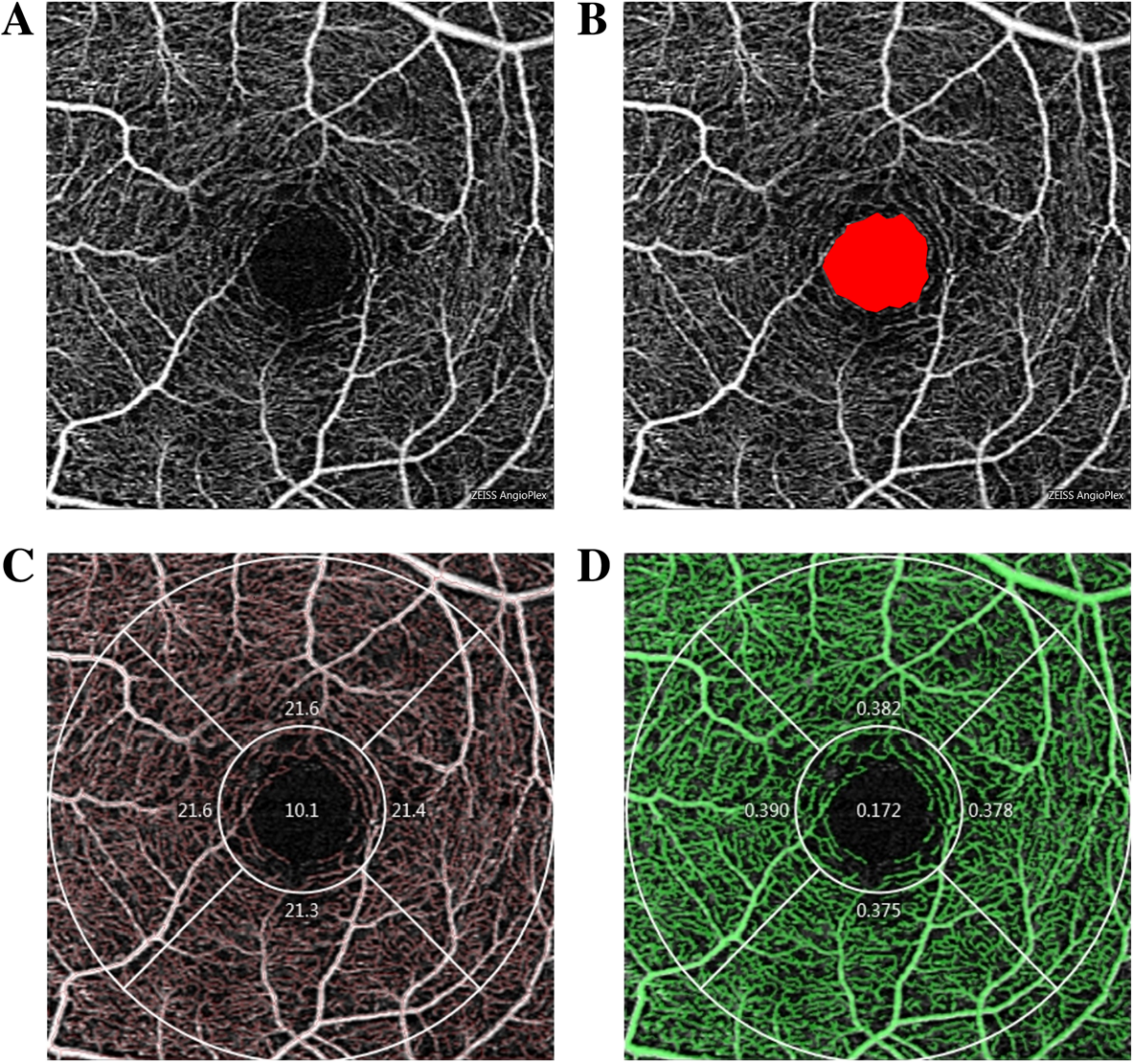
The 3 × 3 mm optical coherence tomography angiography image of the macular region of the retina (**a**) OCTA image showing the detailed structure of superficial retinal vessels (**b**) OCTA image showing the FAZ area (**c**) OCTA image showing the measurement of vascular density in the macular region (**d**) OCTA image showing the measurement of perfusion density in the macular region

OCTA images of the macula were obtained using a Cirrus high-definition OCT prototype with AngioPlex (Carl Zeiss Meditec, Dublin, CA, USA). The macula was imaged using a 3 × 3 mm scan pattern. Tracking technology was applied to reduce the effect of motion artifacts. Only high-quality images with signal strengths over 8 were included for analysis. Parameters to evaluate the superficial retinal vessels (from inner boundary membrane layer to the inner plexus layer), including foveal avascular zone (FAZ), vascular density and perfusion density, were calculated using the manufacturer’s angiometric software (Fig. 1). Vascular density is the linear length of vessels divided by the selected area. Perfusion density represents the area of vessels distribution divided by the selected area. Although both eyes were eligible for our study, only right eye was selected in the final data analysis.

### Statistical analysis

The Statistical Package for Social Sciences version 22.0 for Windows (SPSS Inc., IBM Corp., Chicago, IL, USA) was used for data analysis. The Levene test was selected to assure the variance homogeneity. The Kolmogorov-Smirnov test was performed to check the normality of the data distributions. Chi-square tests were used to analyze the categorical variables. For comparisons of normally distributed data among three groups, the one-way analysis of variance was chosen, and least square difference (LSD) test was subsequently performed for group comparisons; otherwise, a Kruskal-Wallis test was adopted, and then a Mann-Whitney U test was used for group comparisons. Pearson’s correlation coefficients were calculated to evaluate the relationship between different clinical parameters. The receiver operating characteristic (ROC) curve and the area under curve (AUC) were conducted to assess the diagnostic capability of different parameters in TAO. Sensitivities at fixed specificities (85 and 95%) were calculated for different parameters. A *P* value of < 0.05 was considered statistically significant in our analysis.

## Results

A total of 82 eligible subjects were enrolled, including 20 active TAO patients, 33 inactive TAO patients, and 29 healthy participants. The basic characteristics of all participants are shown in Table 1. There were no significant differences in age, sex distributions, AL and CCT among the three groups (*P* = 0.339, *P* = 0.121, *P* = 0.100, *P* = 0.633, respectively). As expected, the proptosis in active TAO patients (21 ± 3) was highest, followed by inactive TAO patients (18 ± 3) and healthy controls (16 ± 2) (*P* < 0.001). Active TAO patients also had higher IOP than the other two groups (*P* < 0.001).

**Table 1:** The basic characteristics of the study population

	Active TAO	Inactive TAO	Normal	*P* value
Age (years)	43.5 ± 11.5	39.3 ± 11.3	38.7 ± 12.5	0.339
Sex (F/M)	12/8	27/6	24/5	0.121
Proptosis (mm)	21 ± 3	18 ± 3	16 ± 2	< 0.001
IOP (mmHg)	22.6 ± 4.3	16.5 ± 3.3	15.4 ± 2.6	< 0.001
AL (mm)	23.53 ± 1.28	24.08 ± 1.01	24.20 ± 1.08	0.100
CCT (μm)	541 ± 27	540 ± 27	534 ± 30	0.633

The traditional OCT analysis results are summarized in Table 2. The global average RNFL thickness was significantly different among the three groups (*P* < 0.001), post hoc pairwise comparisons revealed that active TAO patients had thinner RNFL thickness than the other two groups (*P* < 0.001, *P* < 0.001). Similar results were obtained when comparing the temporal and inferior RNFL thickness (*P* = 0.001, *P* = 0.001), while no significant differences were observed in superior and nasal RNFL thickness (*P* = 0.458, *P* = 0.117). With regard to CT in the macular region, TAO patients, no matter active or inactive, both had significantly higher CT than healthy individuals (all *P* < 0.05). However, no significant differences were detected between active and inactive TAO patients (all *P* > 0.05).

**Table 2:** OCT analysis results in different study groups (μm)

	Active TAO	Inactive TAO	Normal	*P* value	P1	P2	P3
**RNFL thickness**							
Average	92 ± 7	101 ± 8	102 ± 8	< 0.001	< 0.001	< 0.001	0.999
Superior	116 ± 15	121 ± 18	122 ± 16	0.458			
Temporal	72 ± 10	84 ± 13	83 ± 13	0.001	0.002	0.004	0.991
Inferior	117 ± 11	133 ± 16	132 ± 15	0.001	0.001	0.004	0.932
Nasal	63 ± 7	68 ± 10	67 ± 9	0.117			
**Choroidal thickness**							
Average	257 ± 37	260 ± 42	218 ± 32	< 0.001	0.795	< 0.001	< 0.001
Subfoveal	304 ± 41	299 ± 45	258 ± 38	< 0.001	0.676	< 0.001	< 0.001
Superior	270 ± 33	274 ± 42	216 ± 30	< 0.001	0.753	< 0.001	< 0.001
Temporal	261 ± 42	265 ± 41	223 ± 38	< 0.001	0.752	0.002	< 0.001
Inferior	251 ± 36	264 ± 41	216 ± 30	< 0.001	0.193	0.001	< 0.001
Nasal	235 ± 50	241 ± 45	203 ± 38	0.004	0.655	0.016	0.001

P1: *P* value for the comparison group between active TAO and inactive TAO

P2: *P* value for the comparison group between active TAO and normal controls

P3: *P* value for the comparison group between inactive TAO and normal controls

The evaluation of the superficial retinal vessels measured by OCTA are summarized in Table 3. The mean area of FAZ in the active TAO group was 0.36 ± 0.09 mm^2^, which was significantly larger than the other two groups (*P* = 0.045, *P* = 0.001). In contrast, there was no significant difference between the inactive TAO group and control group (*P* = 0.130). However, the inactive TAO group had significantly higher vascular density than the other two groups (all *P* < 0.05), while there were no significant differences between the active TAO group and normal group (all *P* > 0.05). With regard to the perfusion density, significant differences were observed in the temporal and inferior areas (*P* = 0.045, *P* = 0.001), as well as the average values (*P* = 0.032). But the pairwise comparison results were not completely consistent.

**Table 3:** OCTA analysis results in different study groups

	Active TAO	Inactive TAO	Normal	*P* value	P1	P2	P3
**FAZ area (mm**^**2**^**)**	0.36 ± 0.09	0.31 ± 0.08	0.28 ± 0.08	0.006	0.045	0.001	0.130
**Vascular density (mm**^**−1**^**)**							
Superior	21.1 ± 1.5	22.0 ± 1.1	21.4 ± 1.8	0.063			
Temporal	20.3 ± 1.6	21.5 ± 1.1	20.7 ± 2.1	0.026	0.012	0.485	0.044
Inferior	21.0 ± 1.4	21.9 ± 1.3	20.6 ± 1.8	0.004	0.027	0.481	0.001
Nasal	21.2 ± 1.4	22.0 ± 1.2	21.5 ± 1.9	0.148			
Average	21.0 ± 1.1	21.9 ± 0.9	21.0 ± 1.7	0.005	0.003	0.691	0.014
**Perfusion density**							
Superior	0.380 ± 0.029	0.393 ± 0.021	0.381 ± 0.031	0.097			
Temporal	0.367 ± 0.030	0.386 ± 0.020	0.368 ± 0.041	0.045	0.016	0.508	0.092
Inferior	0.378 ± 0.025	0.387 ± 0.028	0.360 ± 0.028	0.001	0.222	0.025	< 0.001
Nasal	0.384 ± 0.034	0.390 ± 0.022	0.385 ± 0.027	0.619			
Average	0.382 ± 0.033	0.389 ± 0.016	0.372 ± 0.026	0.032	0.309	0.181	0.009

P1: *P* value for the comparison group between active TAO and inactive TAO

P2: *P* value for the comparison group between active TAO and normal controls

P3: *P* value for the comparison group between inactive TAO and normal controls

**Fig. 2:**
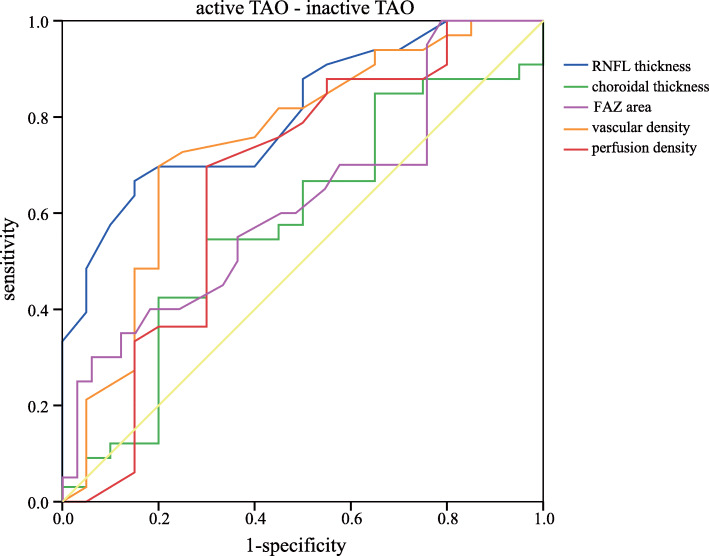
ROC curves for different parameters to discriminate active TAO from inactive TAO eyes

**Fig. 3:**
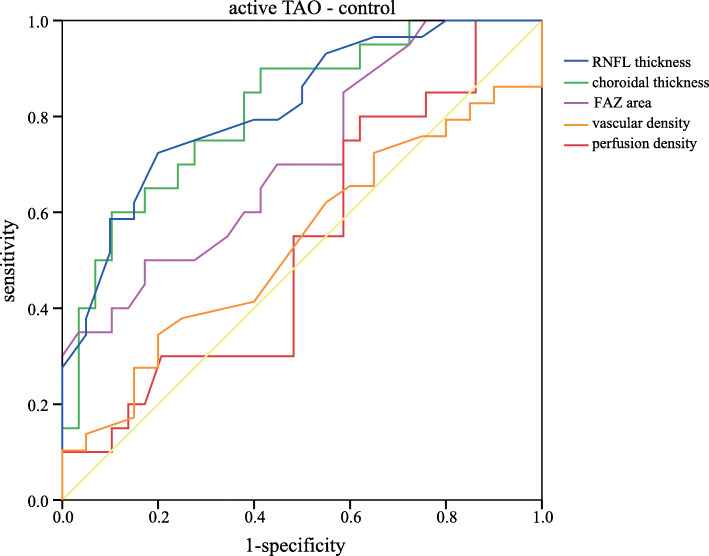
ROC curves for different parameters to discriminate active TAO from normal eyes

**Fig. 4:**
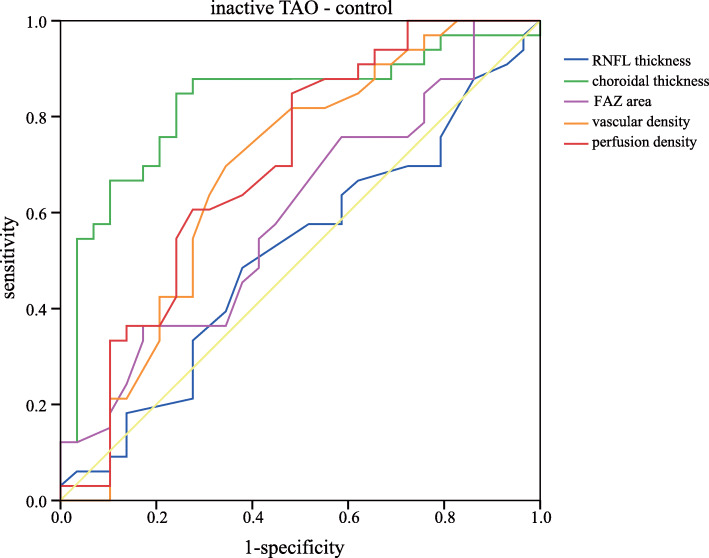
ROC curves for different parameters to discriminate inactive TAO from normal eyes

To determine the potential influencing factors associated with these above parameters, Pearson’s correlation coefficients were calculated with the clinical variables of all the 82 eyes (Table 4). The FAZ area was positively correlated with IOP (r = 0.274, *P* = 0.013), while it was negatively correlated with axial length (*r* = − 0.344, *P* = 0.002). The vascular density and perfusion density were not significantly correlated with different clinical variables (all *P* > 0.05). Moreover, to determine whether these parameters can be used in TAO diagnosis, ROC curves were generated (Table 5). Table 6 showed sensitivities at fixed specificities and their cut-off values. The AUC analysis indicated that RNFL thickness had modest diagnostic power in active TAO/inactive TAO and active TAO/normal subgroups (AUC = 0.804, *P* < 0.001; AUC = 0.818, *P* < 0.001). Comparisons of CT yielded ROC curve areas of 0.814 for active TAO/normal and 0.828 for inactive TAO/normal (*P* < 0.001, *P* < 0.001). In contrast, FAZ area only exhibited a significant discriminatory power in active TAO/normal comparison (AUC = 0.711, *P* = 0.013). The vascular density and perfusion density also showed significant diagnostic ability in active TAO/inactive TAO and inactive TAO/normal subgroups (all *P* < 0.05), but exhibited a poor discriminatory power. Figures 2, 3 and 4 showed the detailed ROC curves for different subgroups.

**Table 4:** Associations between OCTA parameters and clinical variables

	FAZ area		Average vascular density		Average perfusion density	
	r	*p*	r	*p*	r	*p*
Age	0.136	0.224	−0.114	0.308	−0.076	0.500
Proptosis	0.152	0.173	0.051	0.652	0.122	0.276
IOP	0.274	0.013*	0.008	0.994	0.195	0.079
AL	−0.344	0.002*	0.118	0.292	0.025	0.821
CCT	0.093	0.406	−0.010	0.927	−0.057	0.610
Average RNFL	−0.070	0.531	0.169	0.129	0.113	0.312
Average CT	0.124	0.267	0.043	0.700	0.184	0.099

* indicated significant difference with *P* < 0.05

**Table 5:** Area under curve with 95% confidence interval in different subgroups

	Active TAO/inactive TAO		Active TAO /Normal		Inactive TAO /Normal	
	AUC (95%CI)	*P*	AUC (95%CI)	*P*	AUC (95%CI)	*P*
RNFL thickness	0.804 (0.704,0.904)	< 0.001	0.818 (0.701,0.935)	< 0.001	0.514 (0.368,0.660)	0.849
Choroidal thickness	0.569 (0.424,0.714)	0.353	0.814 (0.693,0.935)	< 0.001	0.828 (0.719,0.936)	< 0.001
FAZ area	0.617 (0.468,0.766)	0.117	0.711 (0.564,0.858)	0.013	0.595 (0.453,0.737)	0.202
Vascular density	0.743 (0.610,0.877)	0.001	0.534 (0.371,0.696)	0.692	0.682 (0.544,0.821)	0.014
Perfusion density	0.663 (0.506,0.820)	0.029	0.533 (0.367,0.699)	0.699	0.699 (0.565,0.833)	0.007

**Table 6:** Sensitivity and specificity of different parameters in different subgroups

	active TAO/inactive TAO			active TAO/normal			inactive TAO/normal		
	Cut-off	Sen	Spe	Cut-off	Sen	Spe	Cut-off	Sen	Spe
RNFL thickness	99	0.667	0.850	98	0.724	0.800	103	0.485	0.621
Choroidal thickness	258	0.545	0.700	250	0.103	0.400	241	0.848	0.759
FAZ area	0.28	0.758	0.300	0.35	0.172	0.500	0.35	0.364	0.828
Vascular density	21.6	0.697	0.800	21.6	0.345	0.800	21.6	0.697	0.655
Perfusion density	0.381	0.697	0.700	0.381	0.483	0.700	0.376	0.848	0.517

*Sen* Sensitivity, *Spe* Specificity

## Discussion

OCTA is a new imaging technology that provides noninvasive fundus angiography, which works without contrast medium and avoids allergies and various contraindications. OCTA relies on the intrinsic motion of the fundus vasculature network to separate stationary structures to identify the blood flow. OCTA can also provide a 3D partition by comparing 2D images taken by indocyanine green angiography and fluorescein fundus angiography [18], which avoids artifacts and limitations such as a limited measurement time window and discomfort during inspection. OCTA is reliable, noninvasive, efficient, high quality and safe for fundus vascular imaging [18, 19]. Fundus perfusion depends on the orbital blood supply, and patients with TAO show orbital perfusion changes caused by pathological changes in orbital tissues. A previous study found certain hemodynamic changes in the ocular vasculature under Doppler imaging, and the condition in the ocular vasculature improved after orbital decompression [9]. Although color Doppler imaging has been widely used in vessel inspection, after certain ocular vasculature changes were detected, fundus perfusion changes can hardly be observed by Doppler imaging. Therefore, OCTA would be a perfect choice for further inspection to evaluate the status in fundus vessels.

In our present study, significant thinner RNFL thickness accompanied by higher IOP level was observed in active TAO patients, and the most affected RNFL quadrants were temporal and inferior quadrants. The superficial retinal vascular plexus may be the capillary network primarily responsible for RNFL nourishment. In our study, the macular superficial vessel density remained unchanged in active TAO patients while the thinning of RNFL was observed, suggesting that losses in the peripapillary RNFL in active TAO patients might have been influenced by factors other than retinal microcirculation. Active TAO patients often suffer from secondary compressive IOP rise, thus leading to RNFL defects [20, 21]. The enlargement of extraocular muscles and increase of orbital soft tissue volume could cause direct compression of the optic nerve, which also contribute to dysthyroid optic neuropathy. Earlier detection of RNFL thinning would suggest the presence of optic neuropathy, indicating its use in the evaluation of this disease profile.

Significant greater CT was observed in active and inactive TAO eyes as compared to the normal eyes. Çalışkan et al. [6] found the subfoveal CT in active TAO patients was significantly greater than those with inactive TAO or healthy individuals, even after adjusting for age, axial length and IOP. Similar results were observed in another study conducted by Özkan et al. [22]. Besides, Yu et al. [11] also identified increased CT in TAO patients at different locations in the macular region. Theoretically, one possible explanation for the choroidal variations might be the venous obstruction and congestion, caused by reduce orbital venous drainage, which was the result of increased retrobulbar pressure [22, 23]. Furthermore, the hyperdynamic cardiovascular state of hyperthyroidism could induce an increase in cardiac output, possibly affecting the choroidal perfusion [24].

OCTA has been widely used to analyze the detailed characterization of the retinal and choroidal vasculature in the macular and peripapillary regions [25–27]. Due to the limitations of the analysis software, only superficial vascular plexus in the macular region could be quantitatively analyzed in our study. The FAZ area is a capillary-free area in the central macula that serves as the most sensitive part of the retina. In our study, the FAZ area was significantly enlarged in active TAO patients. But we didn’t detect disintegrity of the vascular arcades surrounding the FAZ area. Previous studies reported that the enlargement of FAZ area more objectively supported the findings of capillary nonperfusion [28, 29]. However, our findings suggested the superficial vascular plexus was increased in inactive TAO patients. The FAZ area measurements showed high reproducibility and repeatability, but the real data vary in different studies. Several possible confounding factors, such as age, sex, spherical equivalent, and axial length, may influence the size of FAZ area [30, 31]. In our study, the FAZ area was positively correlated with IOP (*r* = 0.274, *P* = 0.013), while it was negatively correlated with axial length (*r* = − 0.344, *P* = 0.002), which might give a partial explanation.

The macular vascular density and perfusion density of the superficial layer were quantitatively evaluated in TAO patients. Inactive TAO patients had significantly higher vascular density than that in active TAO and controls. With regard to perfusion density, the pairwise comparison results were not completely consistent. Collectively, the data analysis revealed that inactive TAO patients seemed to have greater perfusion density. In previous studies, Ye at al [32]. reported that active TAO patients presented with an increased retinal microvascular density. Akpolat et al. [33] demonstrated that the temporal and nasal parafoveal vessel density was significantly higher in inactive TAO group, which was consistent with our results. However, Tehrani et al. [34] found active and inactive TAO patients both had significantly lower superficial vessel density in the nasal parafoveal sector. Mihailovic et al. [35] also showed inactive TAO patients had decreased vessel density in the superficial OCT angiogram. In these studies, the normal controls were not totally matched to the study population on several nonexperimental factors, such as age, sex, and axial length, which might at least partially explain the discrepancy. Besides, technical differences and variations in patient status, such as inactive or active TAO state or moderate-to-severe status, may also contribute to the incongruity. Further studies with more rigorous design are desiderated to explore the alterations in TAO patients.

These retinal and choroidal changes might be correlated with variations in orbital blood flow. Doppler imaging of orbital vessels revealed that the resistance index (RI) in the ophthalmic artery (OA) was decreased inactive TAO patients, but systolic velocity remained unchanged, suggesting increased blood flow in OA in inactive TAO patients. The RI in the central retinal artery (CRA) was increased, and the velocity and RI in superior ophthalmic vein (SOV) showed no difference compared with that in the control group [9]. The increased blood supply in the ocular vasculature may partly explain the increased vessel density. Walasik-Szemplińska D et al. [9] found that RI in OA decreased in active TAO. Velocity and RI were increased in CRA, while increased RI and decreased velocity were detected in SOV, indicating circulatory disorder in the ocular vasculature. Reverse flow was also observed in SOV, indicating severe stasis in SOV, which usually correlated with enlarged extraocular muscles. The SOV was considered to play important roles in the inflammatory stage in TAO, and recent studies have demonstrated blood flow reduction in the SOV during active TAO, indicating orbital circulation disorder was resulted from a total effect of increasing venous pressure and high RI, which was caused by elevated intraorbital pressure. Autoimmune inflammation in orbital tissues, including interstitial tissues, orbital fat and extraocular muscles, was the main cause of the high intraorbital pressure [3]. Moreover, Onaran et al. [36] observed a reduction in SOV flow among patients after orbital decompression along with the disappearance of the reverse flow. Therefore, we proposed that vascular physiological changes and elevated intraorbital pressure caused by the direct effect of autoimmune inflammation on ocular vessel and orbital tissues lead to variations in fundus blood flow in active TAO, along with effects on RNFL thickness, CT, FAZ, vessel density and perfusion density, as observed in our study.

In the ROC analysis, OCT-derived RNFL thickness and choroidal thickness showed apparent diagnostic ability in TAO, which were consistent with previous studies [10, 18]. The FAZ area, vascular density and perfusion density also exhibited a significant discriminatory power to distinguish between TAO patients and controls. Ye at al [32]. also showed high diagnostic power to differentiate active TAO patients from normal controls, with AUC of 0.97 for superficial retinal density and AUC of 0.8 for deep retinal density. We hypothesize that these parameters may have the predictive value in the diagnosis of TAO. In addition, non-invasive measurements of these parameters are easily accepted by the patients. Clearly, these parameters were poor markers, further investigations are needed to substantiate these findings in a much larger cohort.

As a preliminary study, our present findings have several limitations. First, this was a cross-sectional study without follow-up data, which prevented us to correlate the vascular changes with the disease progression. Second, the morphology of the superficial retinal vessels is not equal to the hemodynamic changes, which may limit our understanding of the pathogenesis. Third, analysis of the retinal vessel parameters was limited to the superficial layer due to the limitation of analysis software, further investigations of deep retinal vessels should be performed to better demonstrating the retinal vessel variations. Fourth, the sample size was relatively small due to the rigorous selection standards. Moreover, the study group included in the current analysis were TAO patients. Dysthyroid patients in the absence of TAO were not enrolled, which may also limit the interpretation of the results to some extent. Nevertheless, our results indicated retinal and choroidal variations in TAO patients, further researches are highlighted to supplement and extend these preliminary results.

## Conclusion

TAO patients had significant variations in RNFL thickness, choroidal thickness, FAZ area and superficial retinal vessels. These parameters appeared to be potential adjuncts in the evaluation of TAO patients.

## Data Availability

All data generated or analyzed to support the findings of this study is available in the paper without restriction. The raw data during this study is available from the corresponding author on reasonable request.

## Abbreviations

TAO: Thyroid-associated ophthalmopathy
OCT: Optical coherence tomography
RNFL: Retinal nerve fiber layer
CT: Choroidal thickness
OCTA: Optical coherence tomography angiography
CAS: Clinical activity score
IOP: Intraocular pressure
CCT: Central corneal thickness
AL: Axial length
FAZ: Foveal avascular zone
ROC: Receiver operating characteristic
AUC: Area under curve
RI: Resistance index
OA: Ophthalmic artery
CRA: Central retinal artery
SOV: Superior ophthalmic vein
